# In Situ Construction of Thiazole-Linked Covalent Organic Frameworks on Cu_2_O for High-Efficiency Photocatalytic Tetracycline Degradation

**DOI:** 10.3390/molecules30153233

**Published:** 2025-08-01

**Authors:** Zhifang Jia, Tingxia Wang, Zhaoxia Wu, Shumaila Razzaque, Zhixiang Zhao, Jiaxuan Cai, Wenao Xie, Junli Wang, Qiang Zhao, Kewei Wang

**Affiliations:** 1 Department of Chemistry and Chemical Engineering, Shanxi Datong University, Datong 037009, China; jiazf03110088@sxdtdx.edu.cn (Z.J.); 220703001101@sxdtdx.edu.cn (T.W.); 200703001107@sxdtdx.edu.cn (Z.W.); 220703001113@sxdtdx.edu.cn (Z.Z.); 230703001102@sxdtdx.edu.cn (J.C.); 230703001114@sxdtdx.edu.cn (W.X.); wangjunli@sxdtdx.edu.cn (J.W.); 2 School of Chemistry and Chemical Engineering, Huazhong University of Science and Technology, Wuhan 430074, China; 3 Institute of Physical Chemistry, Polish Academy of Sciences, Kasprzaka Street 44/52, 01-224 Warsaw, Poland; srazzaque@ichf.edu.pl

**Keywords:** thiazole-linked covalent organic frameworks, Cu_2_O, heterojunction, photocatalytic degradation

## Abstract

The strategic construction of heterojunctions through a simple and efficient strategy is one of the most effective means to boost the photocatalytic activity of semiconductor materials. Herein, a thiazole-linked covalent organic framework (TZ-COF) with large surface area, well-ordered pore structure, and high stability was developed. To further boost photocatalytic activity, the TZ-COF was synthesized in situ on the surface of Cu_2_O through a simple multicomponent reaction, yielding an encapsulated composite material (Cu_2_O@TZ-COF-18). In this composite, the outermost COF endows the material with abundant redox active sites and mass transfer channels, while the innermost Cu_2_O exhibits unique photoelectric properties. Notably, the synthesized Cu_2_O@TZ-COF-18 was proven to have the heterojunction structure, which can efficiently restrain the recombination of photogenerated electron–hole pairs, thereby enhancing the photocatalytic performance. The photocatalytic degradation of tetracycline demonstrated that 3-Cu_2_O@TZ-COF-18 had the highest photocatalytic efficiency, with the removal rate of 96.3% within 70 min under visible light, which is better than that of pristine TZ-COF-18, Cu_2_O, the physical mixture of Cu_2_O and TZ-COF-18, and numerous reported COF-based composite materials. 3-Cu_2_O@TZ-COF-18 retained its original crystallinity and removal efficiency after five cycles in photodegradation reaction, displaying high stability and excellent cycle performance.

## 1. Introduction

Organic pollutants, such as antibiotics, discharged into wastewater pose significant threats to human health, ecosystems, and the environment [1]. Therefore, effectively removing organic contaminants from wastewater has become imperative. In this context, photocatalytic degradation, an energy-saving and eco-friendly technology, has proven to be an effective strategy for water purification [2]. The key to advancing this technology lies in the development of photocatalytic materials with excellent performance. Metal oxides have remarkable advantages as photocatalysts, ascribed to their effective absorption of light, ease of modification, and doping [3]. Among these, copper(I) oxide (Cu_2_O) is a low-cost and readily available inorganic semiconductor with a narrow band gap of 1.8–2.1 eV. Its broad light absorption spectrum extends well into the visible range, making it highly suitable for photocatalytic and optoelectronic applications [4]. Thus, the unique optical and electrical properties make Cu_2_O a widely used material for photocatalysis [5]. Despite its advantages, Cu_2_O suffers from the rapid recombination of photo-generated electron–hole pairs and photo-corrosion. Its nonporous structure and low surface area also restrict the practical applications in photocatalytic degradation of organic pollutants in wastewater. To enhance its photocatalytic efficiency for practical applications, it is essential to modify the surface of Cu_2_O with a protective material. One effective approach to achieve this is to utilize stable, high-surface-area porous materials, such as covalent organic frameworks (COFs).

COFs are a class of crystalline porous materials formed through the covalent bonding of rigid building blocks. They have gained attention in wastewater treatment due to their inherent advantages, including porosity, large surface areas, and abundant catalytic sites [6,7,8,9]. More remarkably, the tunable pore size, adjustable bandgap, versatile functionalities, and structural diversity of COFs provide tremendous potential as a photodegradation catalyst, enabling task-specific design for water treatment application [10,11,12,13,14]. Among COFs, thiazole-linked COFs (TZ-COFs), containing thiazole rings and fully conjugated structures, exhibit ultrahigh chemical stability in addition to the general characteristics of COFs [15]. They remain stable in harsh conditions, including acidic, basic, oxidative, and reductive environments, thereby extending the photocatalytic lifespan of COFs [16]. Therefore, porous TZ-COFs are an ideal candidate for the protective material of Cu_2_O. In addition, their extended π–conjugation enhances charge carrier transport both in-plane and along the stacking direction [17]. However, most TZ-COFs still fall short of photocatalytic requirements due to low quantum yields, primarily caused by the recombination of photogenerated electron–hole pairs [18].

**Figure 1:**
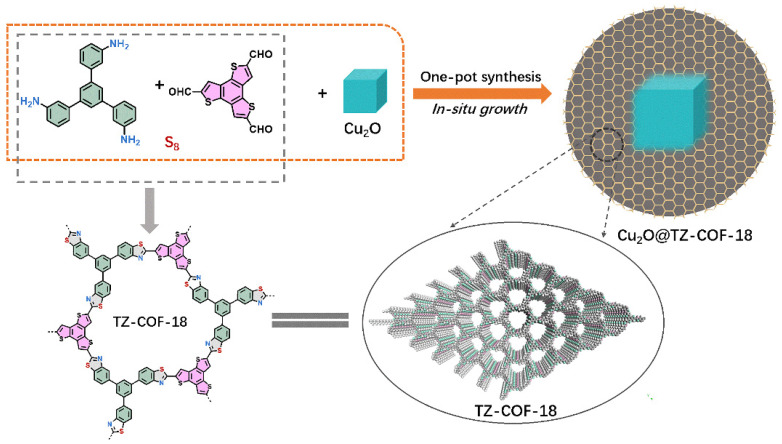
Synthesis of Cu_2_O@TZ-COF-18 and TZ-COF-18.

Considering the advantages of TZ-COFs and Cu_2_O in photocatalysis, modifying the surface of Cu_2_O with TZ-COFs to form a heterojunction is an effective strategy to boost photocatalytic performance. This approach improves the separation efficiency of photo-generated electron–hole pairs by constructing new energy bands [19,20,21,22]. Therefore, we present a simple and feasible strategy for the in situ preparation of thiazole-linked covalent organic frameworks on cubic Cu_2_O, resulting in a series of encapsulated heterojunctions (Cu_2_O@TZ-COF-18, Figure 1). The Cu_2_O was tightly coated by stable TZ-COF, increasing interfacial interactions. Moreover, the stable organic skeleton of the outer COF possesses a large surface area and ordered pores, providing abundant active sites for photocatalytic degradation and channels for the percolation of organic pollutants to access embedded Cu_2_O. The heterojunction prepared by this strategy maximizes the advantages of the Cu_2_O and COF components. More importantly, Cu_2_O@TZ-COF demonstrated superior photocatalytic performance compared to the individual components and physical mixture of Cu_2_O and TZ-COF in the photodegradation of tetracycline (TC).

## 2. Results and Discussion

### 2.1. Characterizations

**Figure 2:**
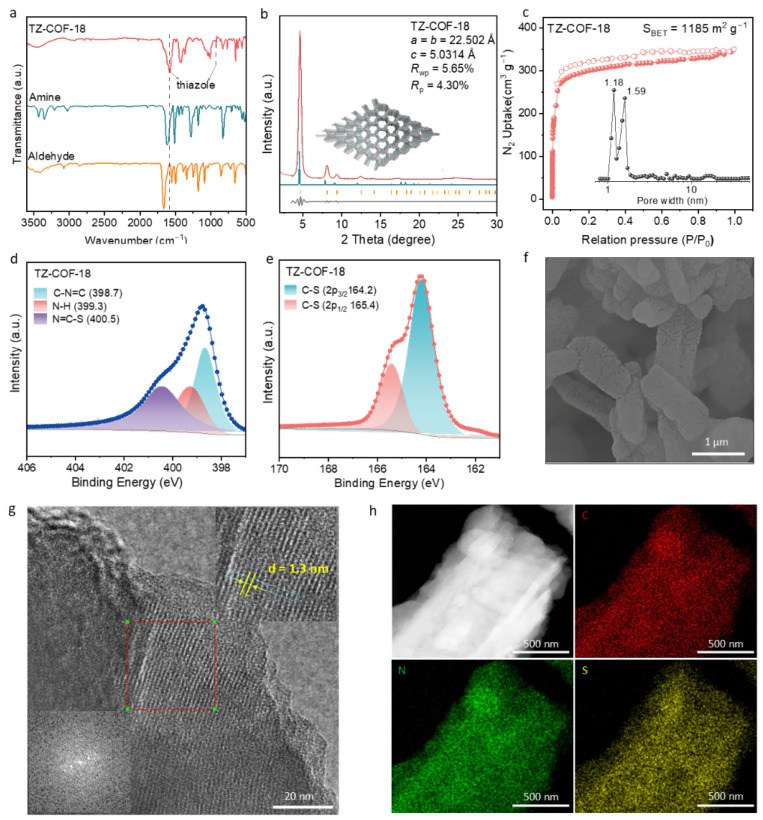
Characterizations of TZ-COF-18. (**a**) FTIR curves for TZ-COF-18 and its monomers. (**b**) PXRD patterns for TZ-COF-18 with eclipsed AA stacking shown parallel to the pore channel along the crystallographic *c* axis (inset). Experimental diffraction pattern (red), profile calculated from Pawley refined (black), residual (gray), and simulated from the structural model (blue). Reflection positions are shown by tick marks. (**c**) N_2_ sorption isotherm and pore size distribution (inset) of TZ-COF-18. XPS spectra of TZ-COF-18 in the region of N 1s (**d**) and S 2p (**e**). (**f**) SEM image of TZ-COF-18. (**g**) TEM image of TZ-COF-18 (insets show a magnified image of the selected area with diffraction points and lattice fringes). (**h**) Element mappings from EDX for TZ-COF-18, showing S, N, and C distributions.

TZ-COF-18 was characterized to determine its structure and physicochemical properties. Fourier-transform infrared (FTIR) spectra of TZ-COF-18 displayed the characteristic absorption peaks of thiazole rings at 1588 and 940 cm^−1^, respectively [23,24], along with the significant weakening of the aldehyde stretching frequency at approximately 1666 cm^−1^ (Figure 2a). The FTIR spectra clearly demonstrated the successful synthesis of thiazole-linked COF via the three-component reaction. The ordered structure of TZ-COF-18 was characterized by powder X-ray diffraction (PXRD). As displayed in Figure 2b, the prominent diffraction peaks at 4.7°, 8.1°, and 9.4° correspond to the (100), (110), and (200) facets, respectively. The experimental result fits well with the simulated PXRD outcome of the eclipsed AA stacking (Figure S1). The Pawley refined unit cell parameters were *a* = *b* = 22.50 Å, *c* = 5.03 Å, *α* = *β* = 90°, and *γ* = 120° (*R*_wp_ = 5.65% and *R*_p_ = 4.30%) (Table S1). The N_2_ adsorption–desorption isotherm was measured to evaluate textural properties, surface area, and pore size distribution. The isotherm exhibited a typical type IV isotherm, revealing typical microporous characteristics with steep N_2_ adsorption at low relative pressure (*P*/*P*_0_ < 0.1), consistent with the pore size distribution plot (Figure 2c). The Brunauer–Emmett–Teller (BET) surface area of TZ-COF-18 was determined to be 1185 m^2^ g^−1^ (Table S2). To validate the oxidation states and chemical environments of the N and S elements in TZ-COF-18, X-ray photoelectron spectroscopy (XPS) measurements were performed (Figure S2). The N 1s spectra deconvoluted into three peaks at 398.7, 399.3, and 400.5 eV, corresponding to –C–N=C, N–H, and N=C–S (thiazole ring) (Figure 2d), while the peaks at 164.2 and 165.4 eV in the S 2p region matched with C–S bonds (Figure 2e) [23]. The scanning electron microscopy (SEM) image displayed rod-like crystallite ranging from several hundred nanometers to a micrometer in length for TZ-COF-18 (Figure 2f and Figure S3). The large crystalline domains were clearly observed in the transmission electron microscopy (TEM) image, along with the lattice spacing of 1.3 nm (Figure 2g and Figure S4). The elements C, N, and S were uniformly distributed throughout the framework of TZ-COF-18, as seen in the energy-dispersive X-ray spectroscopy (EDX) mapping images of TEM (Figure 2h).

The thermal and chemical stabilities of TZ-COF-18 were also investigated. Thermal gravimetric analysis (TGA) of TZ-COF-18 showed decomposition temperature above 400 °C, suggesting high thermostability (Figure S5). For chemical stability testing, TZ-COF-18 was subjected to harsh conditions, including prolonged exposure to boiling water, hydrochloric acid (HCl, 12.0 M), potassium hydroxide (KOH, 12.0 M), sodium methoxide (MeONa, 1.0 M), sodium borohydride (NaBH_4_, 1.0 M), and hydrogen peroxide (H_2_O_2_, 1.0 M). After 48 h treatments, PXRD and FTIR analyses of the recovered TZ-COF-18 showed no significant mass loss, along with retained crystallinity and structural integrity (Figures S6 and S7), providing the evidence for the ultra-high chemical stability of TZ-COF-18. Being an aromatic and fully conjugated organic framework linked by robust covalent bonds, TZ-COF-18 exhibits excellent resistance to acids, bases, and redox agents, and is expected to maintain high stability in actual wastewater containing high concentrations of heavy metal ions, salt ions, and organic pollutants [25].

**Figure 3:**
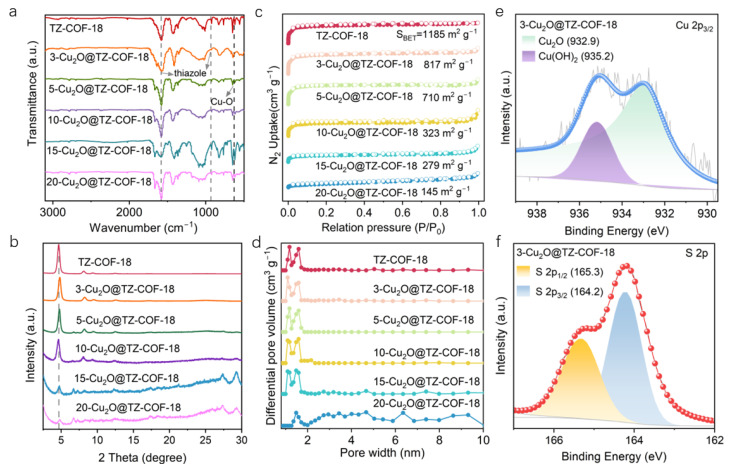
Characterizations of Cu_2_O@TZ-COF-18 composite series. FTIR curves (**a**) and PXRD patterns (**b**) of TZ-COF-18 and Cu_2_O@TZ-COF-18 composites. N_2_ sorption isotherms (**c**) and pore size distributions (**d**) of TZ-COF-18 and Cu_2_O@TZ-COF-18 composites. XPS spectra of 3-Cu_2_O@TZ-COF-18 in the region of Cu 2p (**e**) and S 2p (**f**).

The structure of composites was identified by various techniques. The FTIR spectra of all Cu_2_O@TZ-COF-18 series exhibited characteristic absorption peaks of the thiazole ring at 1588 and 942 cm^−1^, along with the Cu–O peak at 625 cm^−1^ (Figure 3a) [26]. Moreover, the PXRD patterns of all Cu_2_O@TZ-COF-18 series showed the characteristic diffraction peaks consistent with TZ-COF-18, whereas their intensities gradually weakened with the increase of Cu_2_O proportion (Figure 3b). When the amount of Cu_2_O was 20 mg, the main diffraction peak almost disappeared. This could be attributed to the excess Cu_2_O interfering with the ordered growth of TZ-COF-18 during the in situ synthesis. Similarly, all the Cu_2_O@TZ-COF-18 exhibited identical type IV isotherms and pore size distribution as TZ-COF-18 (Figure 3c,d). However, the BET surface area of Cu_2_O@TZ-COF-18 materials decreased with the increase in Cu_2_O proportion. These results clearly manifest the successful formation of the composites composed of Cu_2_O and TZ-COF-18. For structural characterization, we chose 3-Cu_2_O@TZ-COF-18 from the series as a representative sample to analyze its morphology, composition, and crystallinity. To further validate the formation of the thiazole ring and valence state of Cu, XPS measurements were employed for 3-Cu_2_O@TZ-COF-18. The binding energy of Cu 2p_3/2_ at 932.9 eV suggests the presence of Cu_2_O (Figure 3e) [27]. The binding energy at 935.2 eV is assigned to Cu^2+^ species, generated by the inevitable oxidation of Cu_2_O during preparation and storage. The characteristic peaks of the C–S bond in the thiazole rings at 164.2 eV (S 2p_3/2_) and 165.3 eV (S 2p_1/2_) can be seen in the region of S 2p for the 3-Cu_2_O@TZ-COF-18 (Figure 3f).

**Figure 4:**
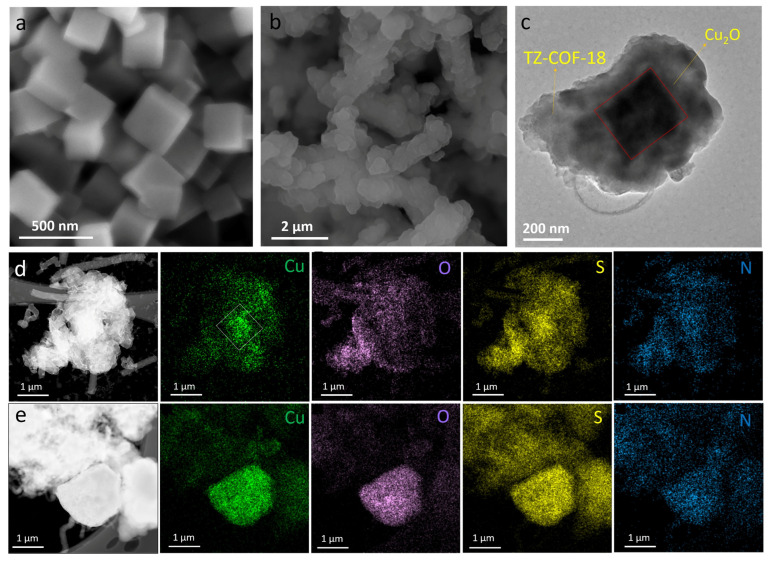
SEM and TEM characterizations of 3-Cu_2_O@TZ-COF-18. SEM images of Cu_2_O (**a**) and 3-Cu_2_O@TZ-COF-18 (**b**). TEM image of 3-Cu_2_O@TZ-COF-18 (**c**). Element mappings from TEM-EDX of full-encapsulated (**d**) and part-encapsulated (**e**) 3-Cu_2_O@TZ-COF-18, showing the distributions of O, S, N, and Cu. The white square in (**d**) indicates that the Cu primarily originates from Cu_2_O.

The morphology of the COF composite series was analyzed using SEM. Herein, we used cubic Cu_2_O (Figure 4a) to fabricate the series of m-Cu_2_O@TZ-COF-18 composites. The SEM image of 3-Cu_2_O@TZ-COF-18 revealed a rod-like morphology composed of irregular nano-sized blocks stacked together (Figure 4b). Further, the TEM images revealed that 3-Cu_2_O@TZ-COF-18 has a capsule-like morphology, where cubic Cu_2_O was encapsulated in TZ-COF-18 (Figure 4c), providing evidence of the successful formation of a heterojunction between Cu_2_O and TZ-COF-18. Moreover, elemental analysis mapping proceeded to verify the spatial distribution of Cu, O, N, and S. As presented in Figure 4d,e, the successful integration of Cu, O, N, and S confirms the proper formation of the heterostructure. Elemental analysis mapping revealed that Cu from Cu_2_O was primarily concentrated at the center of a “capsule”-like heterojunction, while N and S from TZ-COF-18 were uniformly distributed throughout the composite (Figure 4d). Additionally, O was observed across the material, attributed to the presence of oxygen in both Cu_2_O and the unreacted terminal aldehyde groups of TZ-COF-18. To further confirm this, we intentionally selected a defective composite with a partial encapsulated structure for elemental mapping analysis (Figure 4e). As anticipated, the Cu and O elements were predominantly localized on the cubic Cu_2_O. These findings collectively demonstrate that an encapsulated heterojunction between Cu_2_O and TZ-COF-18 was successfully synthesized using an in situ method, through a “one-pot” multicomponent reaction.

### 2.2. Photocatalytic Degradation Performance

**Figure 5:**
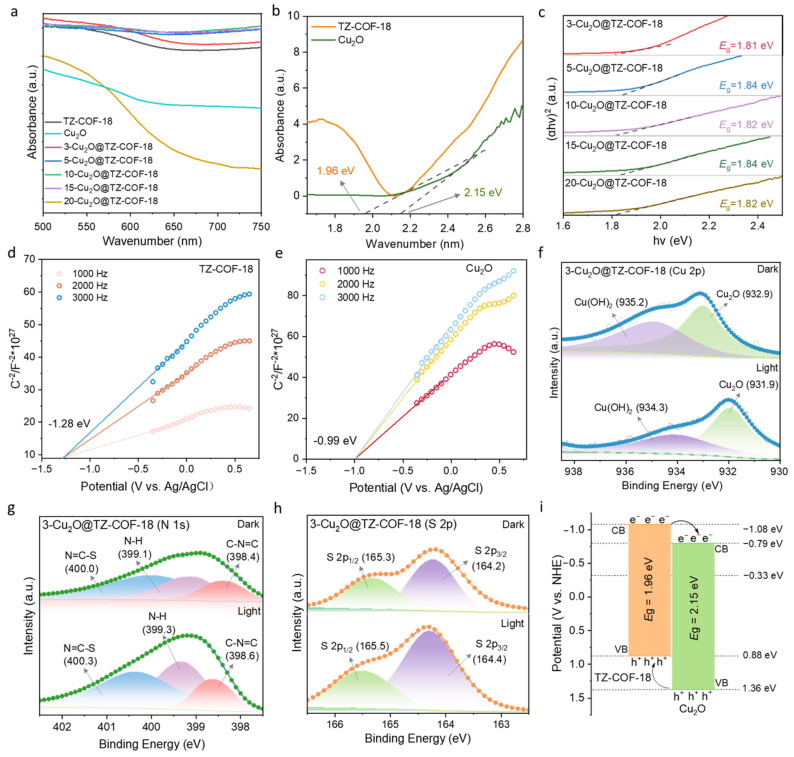
Optoelectronic characterizations of Cu_2_O@TZ-COF-18 composites. (**a**) UV–vis DRS of TZ-COF-18, Cu_2_O, and Cu_2_O@TZ-COF-18 composites. (**b**) Band gaps of TZ-COF-18 and Cu_2_O, calculated using the Tauc plot method. (**c**) Band gaps of Cu_2_O@TZ-COF-18 composites, calculated using the Tauc plot method. Mott–Schottky plots of TZ-COF-18 (**d**) and Cu_2_O (**e**). In situ XPS of 3-Cu_2_O@TZ-COF-18 in dark and light: high-resolution spectra of Cu 2p (**f**), N 1s (**g**), and S 2p (**h**). (**i**) Schematic illustration of the Type II heterojunction between TZ-COF-18 and Cu_2_O.

Capsule-shaped heterojunctions were constructed through the encapsulation of photochemically active Cu_2_O within a robust thiazole-linked COF, thereby improving interfacial charge transfer. Theoretically, optimal band alignment in the heterojunction can significantly enhance photocatalytic efficiency by effectively suppressing the photoinduced electron–hole pair recombination. Therefore, we investigated the photo-absorption characteristics of TZ-COF-18, Cu_2_O, and Cu_2_O@TZ-COF-18 composites using ultraviolet–visible diffuse reflectance spectroscopy (UV-vis DRS) (Figure 5a). Compared with pure TZ-COF-18 and Cu_2_O, the absorption edges of all the Cu_2_O@TZ-COF-18 composites shifted to the ultraviolet region, with the exception of 20-Cu_2_O@TZ-COF-18, implying enhanced light-harvesting ability. The energy band gaps (*E*_g_) of all the composites ranged from 1.81 to 1.84 eV, lower than those of Cu_2_O or TZ-COF-18, further indicating improved absorption of visible light (Figure 5b,c). Mott–Schottky analysis was employed to investigate the electronic properties of TZ-COF-18 and Cu_2_O, particularly their electrochemical behavior and flat-band potential. The flat-band potentials of TZ-COF-18 and Cu_2_O were determined to be −1.28 and −0.99 eV (vs. Ag/AgCl, pH = 6.7), respectively (Figure 5d,e). The conduction band potentials (*E*_CB_) of TZ-COF-18 and Cu_2_O were subsequently derived as −1.08 and −0.79 eV (vs. NHE, pH = 0), using the reported calculation protocol [28]. Combined with the *E*_g_ obtained from UV–vis DRS, the valence band potentials (*E*_VB_) of TZ-COF-18 and Cu_2_O were calculated to be 0.88 and 1.36 eV, respectively. Consequently, the heterojunction structures have been constructed between Cu_2_O and TZ-COF-18, resulting from their matched VB and CB [29,30,31]. In situ XPS measurements were implemented to further confirm the heterojunction types. Compared with the results tested under dark conditions, the characteristic Cu 2p peak of 3-Cu_2_O@TZ-COF-18 moved in the direction of decreasing binding energy, while the binding energies of N 1s and S 2p increased (Figure 5f–h). This illustrated that the electrons transferred from TZ-COF-18 to Cu_2_O under light irradiation, demonstrating the Type II-schemed charge transfer mechanism (Figure 5i) [20]. In short, the optimal band gap and stability of TZ-COF-18 outer shell, combined with its large surface area and numerous pores, provides abundant catalytic active sites and efficient mass transfer channels, thereby facilitating enhanced catalytic activity.

**Figure 6:**
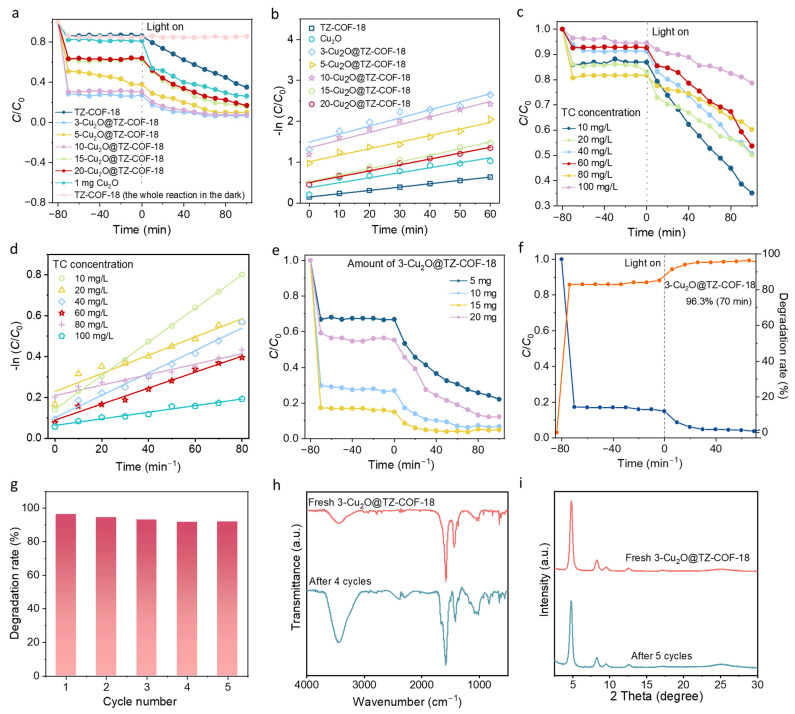
Photocatalytic degradation TC experiments. (**a**) Photocatalytic degradation of TC for TZ-COF-18, Cu_2_O, and the composites within 100 min (Cu_2_O (1 mg), TZ-COF-18 (10 mg) or m-Cu_2_O@TZ-COF-18 (10 mg), TC (10 mg L^−1^), λ > 420 nm, 500 W xenon lamp, 50 mW cm^−2^. The dosages of Cu_2_O (1 mg) and TZ-COF-18 (10 mg) match their respective contents in 10 mg of 3-Cu_2_O@TZ-COF-18; detailed calculations are given in the caption of Figure S8. (**b**) Kinetic fitting curves for TZ-COF-18, Cu_2_O, and the composites in photocatalytic degradation of TC within 60 min. Photocatalytic degradation curves of TC (**c**) and kinetic curves (**d**) for 3-Cu_2_O@TZ-COF-18 (10 mg), with different concentrations of TC. (**e**) Photocatalytic degradation of TC for 3-Cu_2_O@TZ-COF-18 with different amounts, within 100 min. (**f**) Degradation curve and rate for 3-Cu_2_O@TZ-COF-18 (15 mg) in photocatalytic degradation of TC (10 mg L^−1^), within 70 min. (**g**) Cycle testing of 3-Cu_2_O@TZ-COF-18 under the optimal conditions. FTIR (**h**) and PXRD (**i**) spectra of fresh 3-Cu_2_O@TZ-COF-18 and recovered 3-Cu_2_O@TZ-COF-18, after five cycles.

The photocatalytic performances of the heterojunctions (Cu_2_O@TZ-COF-18) were evaluated by studying the degradation of TC under visible-light irradiation. Compared to pristine TZ-COF-18 and Cu_2_O, all five composites exhibited higher degrading efficiency for TC after 100 min of simulated sunlight irradiation (Figure 6a). Among the series of composites, 3-Cu_2_O@TZ-COF-18 (10 mg) had the highest degradation rate, reaching 93.7% after 100 min. In comparison, the degradation rates for the pure Cu_2_O and TZ-COF-18 were only 73.7% and 64.9%, respectively, under the same conditions (Figure S8). Accordingly, the apparent rate constants of all Cu_2_O@TZ-COF-18 materials were higher than pristine Cu_2_O and TZ-COF-18, demonstrating the enhanced photocatalytic performance achieved by composites. Notably, 3-Cu_2_O@TZ-COF-18 exhibited the highest rate constant of 0.0198 min^−1^, which is 2.5 and 1.5 times higher than those of pristine TZ-COF-18 (0.0081 min^−1^) and Cu_2_O (0.0122 min^−1^) (Figure 6b and Figure S8). This highlights the significant improvement in photocatalytic efficiency through the integration of Cu_2_O and TZ-COF-18 in the composite. Based on its superior photocatalytic performance, we selected 3-Cu_2_O@TZ-COF-18 for further investigations, to explore its potential and optimize its properties for catalytic applications. After optimizing the concentration of TC (Figure 6c,d and Figure S9) and the amount of 3-Cu_2_O@TZ-COF-18 used (Figure 6e and Figure S10), the optimal degradation conditions were established as follows: 3-Cu_2_O@TZ-COF-18 (15 mg), TC (10 mg L^−1^), 500 W Xenon lamp, *λ* > 420 nm. Under the optimum conditions, the removal efficiency of TC for 3-Cu_2_O@TZ-COF-18 reached 96.3% after light irradiation for 70 min (Figure 6f), which is significantly higher than those of TZ-COF-18 (51.7%), Cu_2_O (67.9%), and the physical mixture Cu_2_O/TZ-COF-18 (68.5%), respectively (Figure S11). This performance also surpasses that of many previously reported COFs or hybrid materials (Table S3) [29,32,33,34,35,36,37,38,39,40,41,42,43,44,45,46,47]. Compared with commercial P25, which exhibited higher mass efficiency, 3-Cu_2_O@TZ-COF-18 achieved a superior degradation rate within a significantly shorter reaction time, demonstrating its enhanced reaction kinetics and higher ultimate degradation efficiency in practical applications [48,49]. A cycling experiment was conducted under the optimal conditions, to examine the cycling performance of Cu_2_O@TZ-COF-18. After five cycles, the degradation efficiency of 3-Cu_2_O@TZ-COF-18 showed no significant decrease (Figure 6g). Furthermore, the recovered photocatalyst retained its original molecular structure and crystallinity, as confirmed by the FTIR and PXRD measurements (Figure 6h,i), demonstrating the high stability of this heterojunction catalyst throughout the photocatalytic degradation process.

### 2.3. Photocatalytic Degradation Mechanism

**Figure 7:**
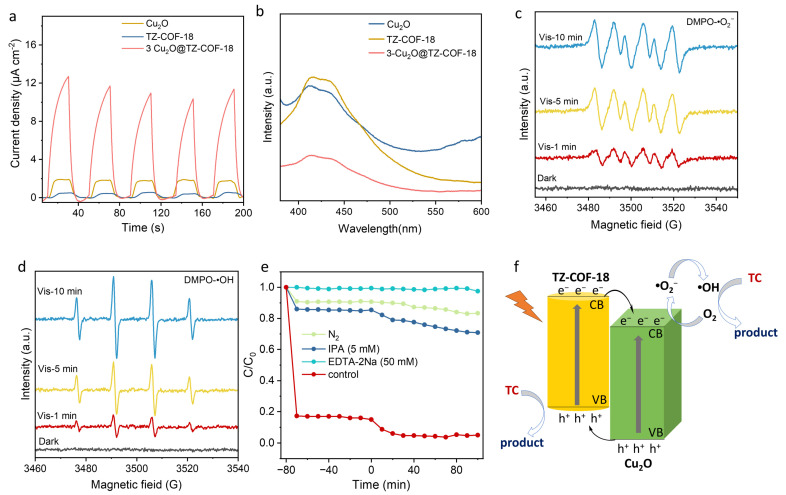
Investigations of photocatalytic degradation mechanism. TPR (**a**) and PL spectra (**b**) of TZ-COF-18, Cu_2_O, and 3-Cu_2_O@TZ-COF-18. EPR spectra of ·OH (**c**) and ·O_2_^−^ (**d**) for 3-Cu_2_O@TZ-COF-18. (**e**) Active-species trapping experiments with 3-Cu_2_O@TZ-COF-18 under visible-light irradiation. (**f**) Proposed photocatalytic degradation mechanism for the 3-Cu_2_O@TZ-COF-18 heterojunction.

To investigate the factors responsible for the improved photocatalytic performance and to uncover the underlying catalytic mechanism, we carried out transient photocurrent response (TPR) measurements (Figure 7a). These measurements revealed that, compared to Cu_2_O and TZ-COF-18, 3-Cu_2_O@TZ-COF-18 exhibited a stronger photocurrent response under light excitation. This indicates that the successful construction of the heterojunction between Cu_2_O and TZ-COF-18 effectively accelerates carrier separation and migration, which is likely a key factor in enhancing its photocatalytic performance. Photoluminescence (PL) measurements were used to further elucidate the efficiency of charge carrier separation (Figure 7b). By analyzing the PL spectra of TZ-COF-18 and Cu_2_O, we observed strong emission signals around 425 nm. However, the signal intensity of 3-Cu_2_O@TZ-COF-18 was remarkably weakened, indicating that the encapsulated heterojunction structure effectively suppresses the charge carrier recombination.

The active species generated during the photocatalytic degradation process were detected and analyzed using electron paramagnetic resonance (EPR) measurements. Generally, the active species, hydroxyl radicals (OH) and superoxide radicals (O_2_^−^) generated during catalysis, play a direct role in the photocatalytic degradation of TC [20]. To capture free radicals, 5-dimethyl-1-pyrroline *N*-oxide (DMPO) was used as a trapping agent. As anticipated, the characteristic signals of DMPO-·OH and DMPO-·O_2_^−^ were observed for 3-Cu_2_O@TZ-COF-18 under the light irradiation, while no such signals were detected in the dark (Figure 7c,d). Moreover, the intensity of these signals enhanced with prolonged illumination time, indicating the generation of large amounts of·OH and·O_2_^−^ on the 3-Cu_2_O@TZ-COF-18 composites under visible light. To test the role of these reactive species during the photodegradation, ethylenediaminetetraacetic acid disodium salt (EDTA-2Na) and isopropanol (IPA) were employed as scavengers for photogenerated holes (h^+^) and ·OH, respectively. The photodegradation was also carried out under a N_2_ atmosphere to remove oxygen, thereby preventing the formation of O_2_^−^. As shown in Figure 7e, the degradation performances of TC significantly decreased after adding the IPA, EDTA-2Na, or under the N_2_ atmosphere, indicating that h^+^, OH, and O_2_^−^ all play crucial roles in the degradation of TC. The comparison of decreasing degradation performance in quenching experiments demonstrates that the influencing sequence of the active species for TC degradation is h^+^ >·O_2_^−^ >·OH.

Based on experimental findings, a plausible mechanism for photocatalytic degradation of TC by 3-Cu_2_O@TZ-COF-18 was proposed, and is illustrated in Figure 7f. Under the solar-light irradiation, both Cu_2_O and TZ-COF-18 were excited, leading to the formation of photogenerated electron–hole pairs. The photo-induced electrons (e^−^) from the CB of TZ-COF-18 were transferred to the CB of Cu_2_O; meanwhile, the holes (h^+^) on the VB of Cu_2_O transferred to the COF, since the VB edge potential of Cu_2_O is more positive than the COF. Consequently, more photogenerated e^−^ and h^+^ accumulated in CB of Cu_2_O and VB of TZ-COF-18, respectively, thereby promoting efficient separation of electron–hole pairs. The accumulated electrons reacted with O_2_ to generate·O_2_^−^, due to the more negative CB potential of Cu_2_O compared to the (O_2_/O_2_^−^) redox potential. Given that the oxidation potentials of H_2_O/OH and OH^−^/OH are higher than the VB potential of TZ-COF-18 (0.88 eV), the OH species detected during the reaction did not originate from the oxidation of h^+^. Instead, they were generated from·O_2_^−^ reacting with h^+^ and e^−^ to form H_2_O_2_, ultimately leading to the production of ·OH through a Fenton-like reaction [40,50]. The generated ·OH and h^+^ can oxidize TC into demethylated and hydroxylated intermediates. Through the collaborative action of ·O_2_^−^, TC was ultimately degraded into small molecules such as CO_2_ and H_2_O [40,51].

## 3. Experimental Section

### 3.1. Synthesis of TZ-COF-18

TZ-COF-18 was synthesized by a multi-component reaction (Figure 1) [15]. 5′-(3-Aminophenyl)-[1,1′:3′,1′’-terphenyl]-3,3′’-diamine (17.60 mg, 0.05 mmol), benzo [1,2-b:3,4-b’:5,6-b’’]trithiophene-2,5,8-tricarbaldehyde (16.50 mg, 0.05 mmol), and sulfur (14.40 mg, 0.45 mmol) were charged into a 10 mL glass tube, along with acetic acid (6 M, 0.10 mL), dimethylsulfoxide (DMSO, 0.05 mL), *o*-dichlorobenzene (0.45 mL), and *n*-BuOH (0.50 mL). The tube was flash frozen in a liquid nitrogen bath, evacuated to an internal pressure of 0.5 Mbar, and flame-sealed. The sealed tube was placed in an oven at 120 °C for 72 h, yielding a yellow-brown solid. The isolated solid was subjected to Soxhlet extraction with toluene and tetrahydrofuran for 48 h, respectively. After drying for 12 h at 80 °C, TZ-COF-18 was obtained as an orange-yellow powder (30.30 mg, 84.4%).

### 3.2. Synthesis of Cu_2_O@TZ-COF-18

To ensure that the TZ-COF was tightly encapsulate around the Cu_2_O to form an encapsulated heterostructure, the cubic-shaped Cu_2_O was prepared via a liquid-phase reduction method [5]. Cu_2_O (m mg), 5′-(3-aminophenyl)-[1,1′:3′,1′’-terphenyl]-3,3′’-diamine (17.60 mg, 0.05 mmol), benzo [1,2-b:3,4-b’:5,6-b’’]trithiophene-2,5,8-tricarbaldehyde (16.50 mg, 0.05 mmol), and sulfur (14.40 mg, 0.45 mmol) were charged into a 10 mL glass tube, along with *o*-dichlorobenzene (0.45 mL), and *n*-BuOH (0.50 mL). After ultrasonication for 5 min until the Cu_2_O was encapsulated by the organic phase, acetic acid (6 M, 0.10 mL) and DMSO (0.05 mL) were added. The subsequent reaction conditions and post-reaction treatment were identical to those of TZ-COF-18. In accordance with the addition of Cu_2_O, the obtained composite was named m-Cu_2_O@TZ-COF-18, where m represents the addition of Cu_2_O (m = 3.0, 5.0, 10.0, 15.0, or 20.0 mg). For 3-Cu_2_O@TZ-COF-18, the isolated product weighed 33.40 mg.

### 3.3. Photocatalytic Degradation Evaluation

Photocatalytic material (10 mg) was added to 80 mL TC aqueous solution with an initial concentration of 10 mg L^−1^. The suspension was stirred in the dark for 70 min to achieve adsorption–desorption equilibrium. Later, the TC suspension was exposed to visible-light irradiation (λ > 420 nm, 500 W Xe lamp), under continuous stirring. The reaction temperature was maintained at 25 °C by circulating cool water. During the irradiation, 2 mL of the reaction mixture was extracted every 10 min. The extracted sample was centrifuged, and the supernatant was analyzed using a 722E UV–vis spectrometer at 357 nm. The incident light power density at the liquid surface was measured to be ~50 mW cm^−2^ with an FZ-A optical power meter (Beijing Shida Photoelectric Technology Co., Ltd.; Beijing; China).

## 4. Conclusions

In summary, an encapsulated heterojunction Cu_2_O@TZ-COF-18 photocatalyst was successfully synthesized via an in situ multicomponent reaction. This strategically engineered composite integrates a robust thiazole-linked COF with a metal oxide semiconductor, offering three key advantages: (1) the TZ-COF-18 protective layer provides a large surface area, abundant redox-active sites, and a hierarchical porous structure to facilitate mass transfer; (2) the heterojunction architecture significantly enhances charge separation efficiency by suppressing the recombination of photogenerated electron–hole pairs, thereby optimizing photocatalytic activity; and (3) the synergistic coupling of COFs and Cu_2_O effectively compensates for their individual limitations, while amplifying their inherent merits in photocatalytic applications. The optimized 3-Cu_2_O@TZ-COF-18 composite exhibited superior photocatalytic performance for tetracycline degradation under simulated sunlight, achieving a degradation rate of 96.3% within 70 min—significantly outperforming the individual components and physical mixture of Cu_2_O and TZ-COF. Systematic investigations, including UV–vis DRS spectroscopy, Mott–Schottky analysis, in situ XPS, TPR, PL spectroscopy, and EPR measurements, revealed that the heterojunction structure effectively suppressed charge carrier recombination, facilitated charge transfer, and promoted the generation of reactive oxygen species (OH and O_2_^−^). The facile construction of the encapsulated heterojunction opens up broad possibilities for developing COF-based photocatalysts, and offers new materials for other photocatalytic processes.

## Data Availability

The original contributions presented in this study are included in the article. Further inquiries can be directed to the corresponding authors.

## Supplementary Materials

The following supporting information can be downloaded at: https://www.mdpi.com/article/10.3390/molecules30153233/s1, Figure S1: PXRD patterns of TZ-COF-18: Experimental (black), calculated with the eclipsed (AA) stacking model (red), staggered (AB) stacking models (blue), and ABC stacking models (green); Figure S2: XPS pattern of TZ-COF-18 in the regions of C 1s, N 1s, O 1s, and S 2p; Figure S3: SEM images of TZ-COF-18; Figure S4: TEM image of TZ-COF-18; Figure S5: TGA of TZ-COF-18 and 3-Cu_2_O@TZ-COF-18; Figure S6: PXRD patterns of TZ-COF-18 measured after 48 h treatment in boiling water, 12.0 M HCl, 12.0 M KOH, 1.0 M NaBH_4_, 1.0 M CH_3_ONa, and 1.0 M H_2_O_2_, respectively; Figure S7: FTIR spectra of TZ-COF-18 measured after 48 h treatment in boiling water, 12.0 M HCl, 12.0 M KOH, 1.0 M NaBH_4_, 1.0 M CH_3_ONa, and 1.0 M H_2_O_2_, respectively; Figure S8: Photocatalytic degradation of TC for TZ-COF-18, Cu_2_O, and the composites within 100 min: Cu_2_O (1 mg), TZ-COF-18 (10 mg), or Cu_2_O@TZ-COF-18 (10 mg); TC (10 mg L^−1^); *λ* > 420 nm; 500 W xenon lamp. Reaction rate constant (a), and degradation curve and tetracycline removal efficiency (b) for 3-Cu_2_O@TZ-COF-18; Figure S9: Reaction rate constants for 3-Cu_2_O@TZ-COF-18 (10 mg) in the photocatalytic degradation of TC at different concentration; Figure S10: Photocatalytic degradation rate constant of 3-Cu_2_O @ TZ-COF-18 with different masses (TC 10 mg L^−1^); Figure S11: Photocatalytic degradation of TC for Cu_2_O/TZ-COF-18 within 70 min: Cu_2_O/TZ-COF-18 was obtained by physical mixing 1.4 mg of Cu_2_O and 13.6 mg of TZ-COF-18; TC (10 mg L^−1^); λ > 420 nm; 500 W xenon lamp. Degradation curve and tetracycline removal efficiency (a) and reaction rate constant (b) for Cu_2_O/TZ-COF-18; Table S1: Crystal structure data for TZ-COF-18; Table S2: Surface area, porosity, and optical gap of COFs; Table S3: Comparison of photocatalytic degradation of TC performance for different photocatalysts reported in the literatures.
