# Supplementary material for: In Situ Construction of Thiazole-Linked Covalent Organic Frameworks on Cu2O for High-Efficiency Photocatalytic Tetracycline Degradation
Source: Molecules. 2025 Aug 1;30(15):3233. doi: 10.3390/molecules30153233 (PMC12348375; doi:10.3390/molecules30153233)
Supplement: Supplementary file 1 [file molecules-30-03233-s001.zip › molecules-3766986-supplementary.pdf]

Supporting Information

In situ Construction of Thiazole-Linked Covalent Organic Frameworks on Cu_2O for High-Efficiency Photocatalytic Tetracycline Degradation

Zhifang Jia,[a,1] Tingxia Wang,[a,1] Zhaoxia Wu,[1,2] Shumaila Razzaque,[3] Zhixiang Zhao,[1] Jiaxuan Cai,[1] Wen-ao Xie,[1] Junli Wang,[1] Qiang Zhao,*[,1] Kewei Wang*[,1]

[1] Department of Chemistry and Chemical Engineering, Shanxi Datong University, Datong, 037009, China. Emails: zhaoqiang@sxdtdx.edu.cn; wangkewei@sxdtdx.edu.cn.

[2] School of Chemistry and Chemical Engineering, Huazhong University of Science and Technology, Wuhan 430074, China.

[3] Institute of Physical Chemistry, Polish Academy of Sciences. Kasprzaka Street 44/52, 01-224 Warsaw, Poland.

[a] *Zhifang Jia and Tingxia Wang contributed equally to this work.*

Table of contents

1. **Instrumentation and Materials**…………………………………………… S2
2. **Synthetic procedures** ………………………………………………………S5
3. **Characterization**………………………………………………………..… S7
4. **Photocatalytic degradation experiment**…………………………….…S14
5. **References**…………………………………………………………………S18

1. Instrumentation and Materials

Benzo[1,2-b:3,4-b':5,6-b'']trithiophene-2,5,8-tricarbaldehyde (BTT22), and 5'-(3-Aminophenyl)-[1,1':3',1''-terphenyl]-3,3''-diamine were purchased from Shanghai Kylpharm Co., Ltd. Acetic acid (AcOH), dimethyl sulfoxide (DMSO), *o*-dichlorobenzene (*o*-DCB), *n*-butyl alcohol (*n*-BuOH), ethanol (EtOH), tetrahydrofuran (THF), and *n*-hexane were purchased from Tianjin Cines Biochemical Technology Co., Ltd. Hydrochloric acid (HCl), sulfur, sodium hydroxide (NaOH), sodium sulfate (Na_2SO_4), potassium hydroxide (KOH), sodium borohydride ($NaBH_4$), and sodium methoxide (CH_3ONa) were purchased from Sinopharm Chemical Reagent Co., Ltd. Ascorbic acid (AA), $CuSO_4$, and tetracycline hydrochloride (TC) were purchased from Shanghai Aladdin Biochemical Technology Co., Ltd. Unless otherwise noted, all reagents were obtained from commercial suppliers and used without further purification.

Fourier-transform infrared spectroscopy (FT-IR). FT-IR spectra were recorded on a Bruker VERTEX 70 spectrometer in the wavenumber range of 4000–400 cm^{-1} using KBr pellets.

Thermogravimetric analysis (TGA). TGA analysis was performed on a PerkinElmer Instruments Pyris 1 TGA thermobalance under a nitrogen flow, heating from room temperature to 800 °C at a rate of 10 °C min^{-1}.

Gas sorption analysis. The surface areas and N_2 adsorption isotherms of samples (at 77.3 K) were obtained using a Micromeritics ASAP 2020 volumetric adsorption analyzer. Before analysis, the samples were degassed at 110 °C for 8 hours under vacuum (10^{-5} bar).

Powder X-ray diffraction (PXRD). Laboratory powder X-ray diffraction (PXRD) data were collected on a Bruker D8 Advance diffractometer equipped with a Cu Kα source (0.1 mm or 0.2 mm divergence slit, static air scatter screen) and a LynxEye XE detector. Kβ radiation was attenuated using a 0.0125 mm Ni filter.

X-ray photoelectron spectroscopy (XPS). XPS spectra in the region of C 1s, O 1s, N 1s, S 2p, and Cu 2p were performed on an ESCALAB MK II spectrometer (VG, UK).

Scanning electron microscopy (SEM). SEM images were obtained using a Hitachi S-4800 cold field emission scanning electron microscope. Samples were prepared by depositing dry powders onto 15 mm Hitachi M4 aluminum stubs using a high-purity carbon adhesive tab, followed by coating with a 2 nm layer of gold using an Emitech K550X automated sputter coater. Imaging was

conducted at a working voltage of 3 kV and a working distance of 8 mm, using a combination of upper and lower secondary-electron detectors.

Transmission electron microscopy (TEM). TEM images were obtained using a Tecnai G20 microscope (FEI Corporation, Hillsboro, OR, USA) operated at an accelerating voltage of 200 kV.

Ultraviolet–visible diffuse reflectance spectroscopy (UV–vis DRS). UV–vis DRS of the polymers were measured on a Shimadzu UV-2550 UV–vis spectrometer, by measuring the absorption of the powders in the solid state.

Transient photocurrent response (TPR) measurements. TPR measurements were carried out on a CHI 760E electrochemical system (Shanghai, China) in a three-electrode cell system. The working electrode was an indium tin oxide (ITO)-coated glass slide. The reference electrode was a Ag/AgCl electrode (-0.35 V vs. standard hydrogen electrode), and the counter electrode was a platinum wire. A binder solution was prepared by mixing 10 μL of Nafion 117 (5% in a mixture of lower aliphatic alcohols and water) with 2 mL of EtOH. This solution was then mixed with the ground polymer (5 mg) and ultrasonicated to disperse the polymer. The resulting mixture (20 μL) was drop-casted onto the ITO glass working electrode (active area: 0.25 cm^2). The sample was dried under reduced pressure for 60 minutes. The three-electrode cell system was purged with N_2 for 30 minutes before measurement. The measurement was performed in an aqueous sodium sulfate solution (0.2 M) by illuminating the back of the ITO working electrode with a solar simulator (1 Sun, class ABA).

Mott–Schottky (MS) analysis. Mott–Schottky curves were measured in a 0.5 M Na_2SO_4 electrolyte solution (pH = 6.7) at alternating current potential frequencies of 1000, 2000 and 3000 Hz.

Fluorescence photoluminescence (PL) spectroscopy. PL spectroscopy was performed using an Edinburgh FLS1000 spectrometer. A certain amount of dry sample was ground and then loaded into a test mold. PL signals were collected at room temperature with an excitation wavelength of 460 nm.

E**lectron paramagnetic resonance (EPR) spectroscopy.** EPR spectra were carried out using the Bruker EMXplus-9.5/12 EPR spectrometer. For the free-radical test, 5,5-dimethyl-1-pyrroline *N*-oxide (DMPO) was used as a spin-trapping reagent to detect $\cdot OH$ or $\cdot O_2^-$.

Photocatalytic degradation procedure. In a typical TC photocatalytic degradation experiment, 10 mg of photocatalytic material was added to 80 mL TC solution (initial concentration: 10 mg L^{-1}).

The reaction suspension was stirred in the dark for 70 min to reach adsorption–desorption equilibrium. Subsequently, the reaction mixture was irradiated under visible light (λ > 420 nm, 500 W, Xe lamp) under continuous stirring. Later, the TC suspension was exposed to visible-light irradiation (λ > 420 nm, 500 W Xe lamp), under continuous stirring. The temperature of the reaction system was maintained at 25 °C by circulating cool water. Every 10 min, 2 mL of solution was extracted, and the solid was removed by centrifugation. The supernatant was collected and analyzed using a 722E spectrometer at 357 nm. The incident light power density at the liquid surface was measured to be ~50 mW cm^{-2} with an FZ-A optical power meter (Beijing Normal University Photoelectric Technology Co., Ltd). The degradation rate of tetracycline was determined according to the formulas 1 and 2:[1]

$$A = \lg(1/T) = Kbc \quad (1)$$

Where A is absorbance, T is transmission ratio (transmittance), K is molar absorptivity, c is the concentration of absorbent material (mol L^{-1}), and b is the absorber thickness.

$$\eta = \frac{C_0 - C}{C_0} \times 100\% = \frac{A_0 - A}{A_0} \times 100\% \quad (2)$$

Where C_0 is the initial concentration of TC (mg L^{-1}); C is the TC concentration at reaction time t (mg L^{-1}); A_0 is the absorbance of TC solution at the initial concentration; and A is the absorbance of TC solution at reaction time t (mg L^{-1}). The correlation curves of -ln (C/C_0) versus t was fitted to obtain -In (C/C_0) = kt. The slope k represents the apparent reaction rate constant. The linear correlation between -In (C_0/C) and t is characteristic of first-order kinetic equations.

2. Synthetic procedures

2.1 General procedure for polymers

Synthesis of TZ-COF-18

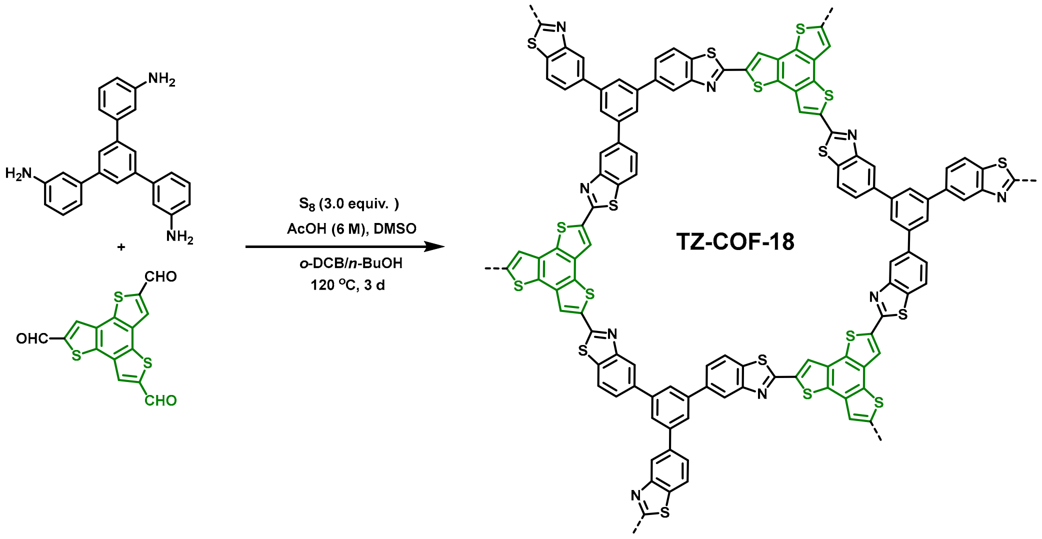

Benzo[1,2-b:3,4-b':5,6-b'']trithiophene-2,5,8-tricarbaldehyde (16.50 mg, 0.05 mmol), 5'-(3-aminophenyl)-[1,1':3',1''-terphenyl]-3,3''-diamine (17.60 mg, 0.05 mmol), and sulfur (14.4 mg, 0.45 mmol) were weighed into a 10 mL glass tube (body length: 18 cm, neck length: 9 cm). AcOH (6 M, 0.10 mL), DMSO (0.05 mL), *o*-DCB (0.45 mL) and *n*-BuOH (0.50 mL) were then added to the mixture. The tube was flash-frozen in a liquid nitrogen bath, evacuated to an internal pressure of 0.5 mbar, and sealed. Upon warming to room temperature, the sealed tube was placed in an oven at 120 °C for 3 days, yielding an orange-yellow solid. The tube was broken at the neck, and the yellow-brown solid was isolated by centrifugation and washed with acetone (3 × 5 mL) and THF (3 × 5 mL). The resulting solid was dried and then subjected to Soxhlet extraction using toluene and THF as the solvent for two days, respectively, to remove the trapped guest molecules. The powder was collected and dried at 80 °C for 12 h, to yield TZ-COF-18 as an orange-yellow powder (30.3 mg, 84.4%).

2.2 General procedure for Cu_2O and Cu_2O@TZ-COF-18

Synthesis of Cu_2O

Cu_2O was prepared by the liquid-phase reduction method, and the synthesis procedures were as follows: a 0.1 M $CuSO_4$ solution (6 mL) was added into 543 mL of deionized water. The solution was warmed to 35°C using a thermostatically heated magnetic stirrer. A 1.0 M NaOH solution (21 mL) was added dropwise to the mixture while stirring, and heating continued. Subsequently, a 0.2

M AA solution (30 mL) was then added dropwise to the mixture, under the stirring and heating conditions. After aging for 3 h, the precipitate was collected by filtration, washed three times with a water/ethanol solution (1:1, v/v), and then dried under vacuum at 40°C overnight to obtain Cu_2O as a yellow-brown powder.

Synthesis of Cu_2O@TZ-COF-18

Benzo[1,2-b:3,4-b':5,6-b'']trithiophene-2,5,8-tricarbaldehyde (16.50 mg, 0.05 mmol), 5'-(3-aminophenyl)-[1,1':3',1''-terphenyl]-3,3''-diamine (17.60 mg, 0.05 mmol), sulfur (14.4 mg, 0.45 mmol), *o*-DCB (0.45 mL) and *n*-BuOH (0.50 mL), and Cu_2O (m mg, where m = 3.0, 5.0, 10.0, 15.0, or 20.0) were weighed into a 10 mL glass tube (body length: 18 cm, neck length: 9 cm). After ultrasonication of the mixture for about 5 minutes until the Cu_2O was encapsulated by the organic phase, AcOH (6 M, 0.10 mL) and DMSO (0.05 mL) was added. The tube was flash-frozen in a liquid nitrogen bath, evacuated to an internal pressure of 0.5 mbar, and sealed. Upon warming to room temperature, the sealed tube was placed in an oven at 120°C for 3 days, yielding a yellow-brown solid. The tube was broken at the neck, and the brown solid was isolated by centrifugation and washed with acetone (3 × 5 mL) and THF (3 × 5 mL). The resulting solid was dried and then subjected to Soxhlet extraction using toluene and THF as the solvent for two days, respectively, to remove trapped guest molecules. The powder was collected and dried at 80 °C for 12 h to yield composite materials as a brown powder. For 3-Cu_2O@TZ-COF-18, the isolated product weighed 33.40 mg.

The photographs of all the synthetic materials are shown below:

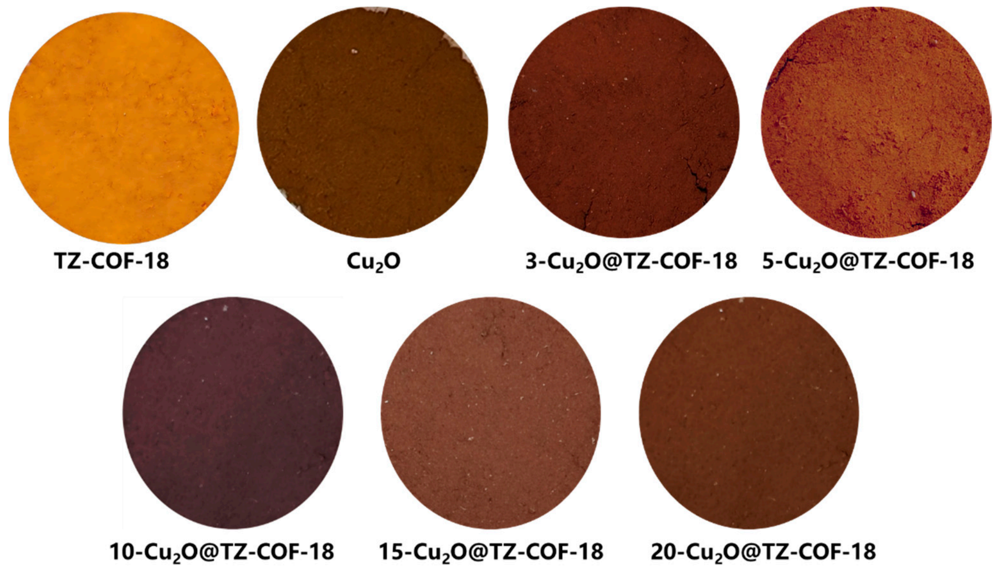

3. Characterization

3.1 Crystal structure data of TZ-COF-18

Table S1. Crystal structure data for TZ-COF-18

TZ-COF-18			
Space group: *P3 (C3-1)*			
$a = b$ = 22.5020 Å, c = 5.03135 Å, $\alpha = \beta$ = 90º, γ =120º			
V = 2206.27 Å^3, R_{wp} = 5.65 %, R_p = 4.30 %			
Atom	*x*	*y*	*z*
C1	1.40282	-1.78051	-0.02621
S2	1.37655	-1.72854	0.11231
C3	1.29341	-1.79048	0.07503
C4	1.2847	-1.84824	-0.04019
N5	1.34711	-1.84294	-0.0962
C6	1.23835	-1.78462	0.15591
C7	1.1716	-1.83719	0.1177
C8	1.15436	-1.89905	-0.00967
C9	1.21449	-1.90429	-0.08609
C10	1.07426	-1.95233	-0.04271
C11	1.0247	-1.92966	-0.04657
C12	1.60246	-1.66984	-0.05929
C13	1.60481	-1.72997	-0.06046
C14	1.53418	-1.68375	-0.05308
C15	1.48235	-1.75347	-0.05439
S16	1.52298	-1.80126	-0.06059
H17	1.24701	-1.73886	0.25275
H18	1.1328	-1.82871	0.19695
H19	1.20698	-1.9499	-0.18005
H20	1.04231	-1.87648	-0.04893
H21	1.52241	-1.64375	-0.04623

3.2 Gas sorption

Table S2. Surface area, porosity, and optical gap of COFs and composite materials

Sample	Cu_2O^a (mg)	$S_{BET}{}^b$ ($m^2\ g^{-1}$)	Predicted $S_{BET}{}^c$ ($m^2\ g^{-1}$)	PV^d ($cm^3\ g^{-1}$)	Optical gap^e (eV)
TZ-COF-18	0	1185	1352	0.541	1.961
3-Cu_2O@TZ-COF-18	3	817	—	0.444	1.813
5-Cu_2O@TZ-COF-18	5	710	—	0.372	1.843
10-Cu_2O@TZ-COF-18	10	323	—	0.314	1.816
15-Cu_2O@TZ-COF-18	15	279	—	0.216	1.842
20-Cu_2O@TZ-COF-18	20	145	—	0.246	1.818

^{a}: initial dosage of Cu_2O in the synthesis of materials. ^{b}: surface area calculated from nitrogen adsorption isotherms at 77.3 K using the BET equation. ^{c}: surface area calculated from theoretical simulation using material studio. ^{d}: total pore volume derived from adsorption isotherms at P/P_0 = 0.095, at 77.3 K. ^{e}: band gap calculation from UV–vis DRS absorption spectrum using Tauc plot analysis.

3.3 PXRD of TZ-COF-18

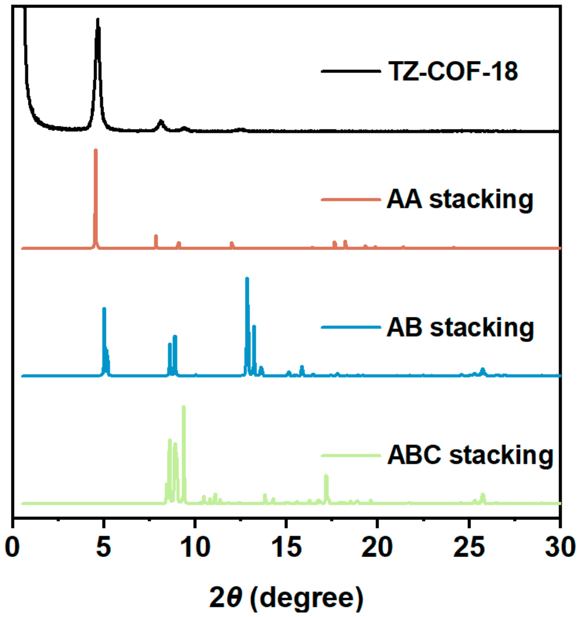

Figure S1. PXRD patterns of TZ-COF-18: experimental (black), calculated with the eclipsed (AA) stacking model (red), staggered (AB) stacking models (blue), and ABC stacking models (green).

3.4 XPS of TZ-COF-18

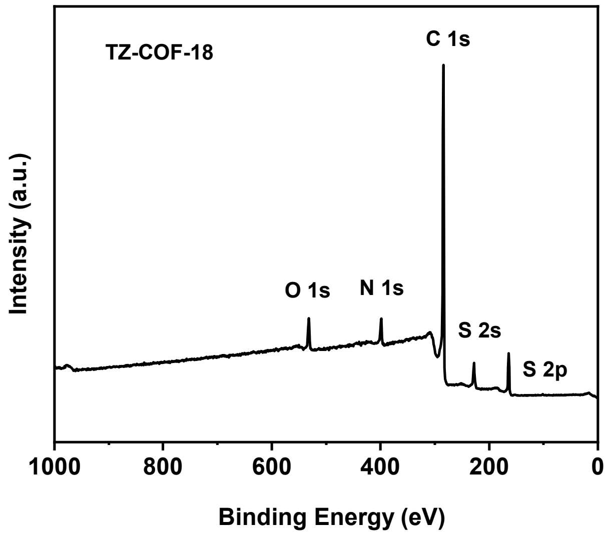

Figure S2. XPS pattern of TZ-COF-18 in the regions of C 1s, N 1s, O 1s, and S 2p.

3.5 SEM and TEM of TZ-COF-18

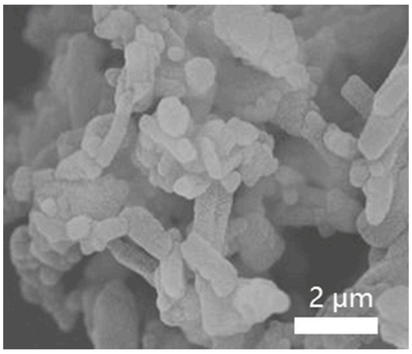

Figure S3. SEM image of TZ-COF-18.

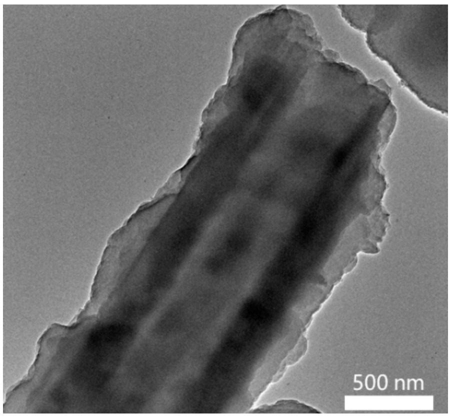

Figure S4. TEM image of TZ-COF-18.

3.6 TGA curves of TZ-COF-18 and 3-Cu_2O@TZ-COF-18

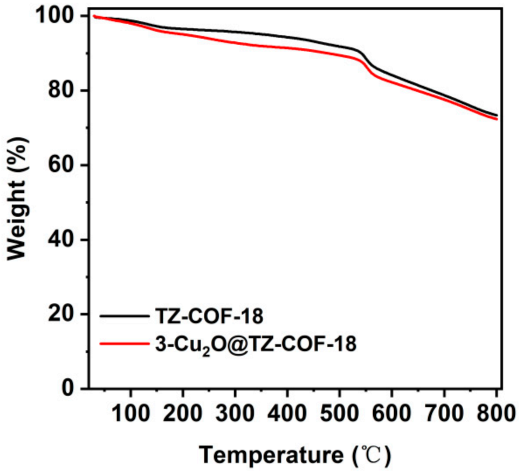

Figure S5. TGA of TZ-COF-18 and 3-Cu_2O@TZ-COF-18.

3.7 Chemical stability of TZ-COF-18

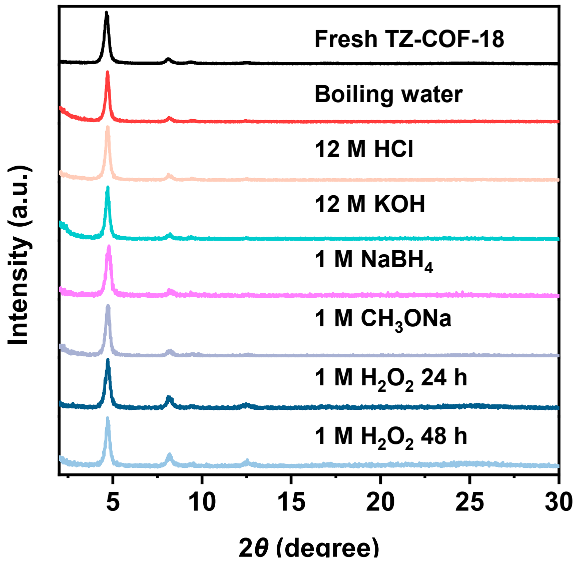

Figure S6. PXRD patterns of TZ-COF-18 measured after 48 h treatment in boiling water, 12.0 M HCl, 12.0 M KOH, 1.0 M $NaBH_4$, 1.0 M CH_3ONa, and 1.0 M H_2O_2, respectively.

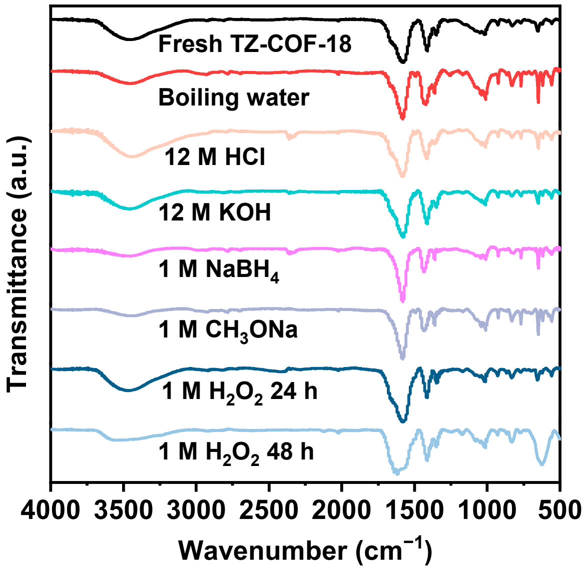

Figure S7. FTIR spectra of TZ-COF-18 measured after 48 h treatment in boiling water, 12.0 M HCl, 12.0 M KOH, 1.0 M $NaBH_4$, 1.0 M CH_3ONa, and 1.0 M H_2O_2, respectively.

4. Photocatalytic degradation experiment

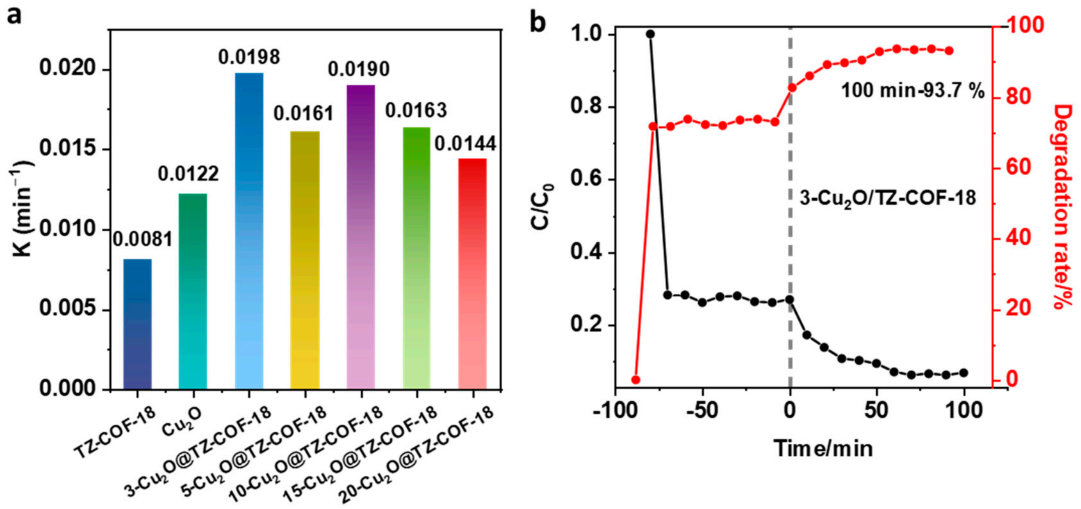

Figure S8. Photocatalytic degradation of TC for TZ-COF-18, Cu_2O, and the composites within 100 min: Cu_2O (1 mg), TZ-COF-18 (10 mg), or Cu_2O@TZ-COF-18 (10 mg); TC (10 mg L^{-1}); $\lambda > 420$ nm; 500 W xenon lamp. Reaction rate constant (a) and degradation curve and tetracycline removal efficiency (b) for 3-Cu_2O@TZ-COF-18. The amounts of Cu_2O (1 mg) and TZ-COF-18 (10 mg) were determined as follows: since 3-Cu_2O@TZ-COF-18 exhibited the best catalytic performance among all the composites, the individual materials—pure Cu_2O and TZ-COF-18—were selected for comparison, with their dosages adjusted to match their respective contents in 10 mg of 3-Cu_2O@TZ-COF-18. Under identical reaction conditions, the isolated mass of TZ-COF-18 was 30.30 mg, while that of 3-Cu_2O@TZ-COF-18 was 33.40 mg. Assuming that the 3 mg of Cu_2O during the synthesis of 3-Cu_2O@TZ-COF-18 remained intact, the mass ratio of Cu_2O to TZ-COF-18 in the composite is approximately 1:10. Therefore, when 10 mg of 3-Cu_2O@TZ-COF-18 was used as the catalyst, the corresponding masses of Cu_2O and TZ-COF-18 present are approximately 1 mg and 10 mg, respectively. Consequently, the comparative experiments employed 1 mg of pure Cu_2O and 10 mg of pure TZ-COF-18.

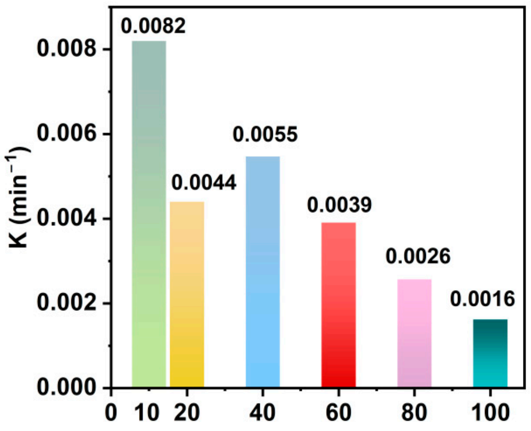

Figure S9. Reaction rate constants for 3-Cu_2O@TZ-COF-18 (10 mg) in the photocatalytic degradation of TC at different concentrations.

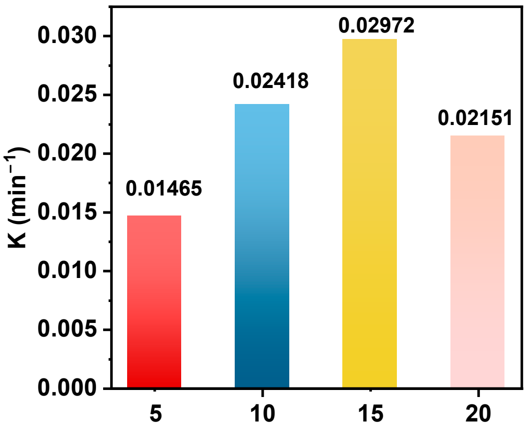

Figure S10. Photocatalytic degradation rate constant of 3-Cu_2O@TZ-COF-18 with different masses (mg) (TC 10 mg L^{-1}).

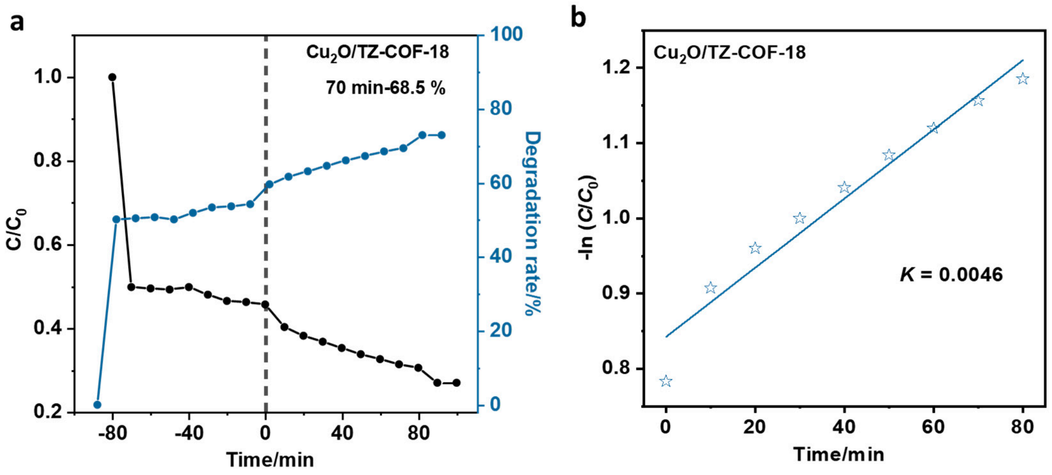

Figure S11. Photocatalytic degradation of TC for Cu_2O/TZ-COF-18 within 70 min: Cu_2O/TZ-COF-18 was obtained by physical mixing of 1.4 mg of Cu_2O and 13.6 mg of TZ-COF-18; TC (10 mg L^{-1}); $\lambda > 420$ nm; 500 W xenon lamp. Degradation curve and tetracycline removal efficiency (a) and reaction rate constant (b) for Cu_2O/TZ-COF-18. The dosages of Cu_2O (1.4 mg) and TZ-COF-18 (13.6 mg) match their respective contents in 15 mg of 3-Cu_2O@TZ-COF-18.

Table S3. Comparison of photocatalytic degradation of TC performance for different photocatalysts reported in the literature

Catalysts	Xenon lamp power (W)	Initial concentration of TC (mg/L)	Catalyst concentration (g/L)	Degradation Rate (%)	Ref.
sp^2c-COF	300	20	0.2	73.5 (90 min)	[2]
COF-DBT	350	10	0.1	95.6 (60 min)	[3]
MOF@COF-2 (Cr)	300	10	0.5	45.86 (120 min)	[4]
π-COF	300	20	0.2	94.8 (90 min)	[5]
ZnO/Cu_2O	300	20	0.01	95.3 (120 min)	[6]
COF@ZIF-2	> 420 nm	15	0.2	90.2 (30 min)	[7]
COF-TzDa/Ag/AgBr/0.4	> 400 nm	10	0.4	80.2 (30 min)	[8]
NH_2-MIL88B/TpPa-1-COF (40)	300	80	0.5	86 (40 min)	[9]
MoS_2/COF (20)	300	20	0.5	85.9 (60 min)	[10]
ZnAgInS/COF(5)	300	60	0.67	90 (60 min)	[11]
COF-Co10	300	10	0.2	77.2 (40 min)	[12]
BAC-2 NCs	300	20	3 mg/100 mL	98.62 (60 min)	[13]
$Co(OH)_2/Cu_2O$	300	10	0.5	98 (30 min)	[14]
$C_{0.7}/Z_{0.3}$	300	50	20 mg/50 mL	84.1 (120 min)	[15]
g-C_3N_4/Cu_2O	300	10	40 mg/40 mL	84 (120 min)	[16]
Bi_2WO_6/C@Cu_2O	300	10	20 mg/50 mL	88 (180 min)	[17]
30% *g*-C_3N_4 nanosheets /Cu_2O	500	30	0.1 g/90 mL	92.1 (100 min)	[18]
P25	500	30	1	< 82.86 (180 min)	[19]
P25	450	100	5 mg/100 mL	30.5 (60 min)	[20]
3-Cu_2O@TZ-COF-18	500	10	15 mg/80 mL	96.34 (70 min)	*This work*

References

[1] H. Wang, X. Quan, Q. Xiong, L. Yin, Y. Tian, J. Zhang, Enhanced performance of β-cyclodextrin modified Cu_2O nanocomposite for efficient removal of tetracycline and dyes: Synergistic role of adsorption and photocatalysis, Appl. Surf. Sci. 621 (2023) 156735.

[2] Z. Hu, Y. Luo, L. Wang, Y. Wang, Q. Wang, G. Jiang, Q. Zhang, F. Cui, Synthesis of pyrene-based covalent organic frameworks for photocatalytic tetracycline degradation, ACS Appl. Polym. Mater. 5 (2023) 9263–9273.

[3] Y. Hou, F. Liu, C. Nie, Z. Li, M. Tong, Boosting exciton dissociation and charge transfer in triazole-based covalent organic frameworks by increasing the donor unit from one to two for the efficient photocatalytic elimination of emerging contaminants, Environ. Sci. Technol. 57 (2023) 11675–11686.

[4] L. Qi, Y. Zhou, J. Qi, Y. Yang, Z. Zhu, C. Xiao, X. Yan, J. Li, Enhanced generation and effective utilization of Cr(V) for simultaneous removal of coexisting pollutants via MOF@COF photocatalysts, ACS EST Eng. 4 (2024) 870–881.

[5] Z. Hu, Y. Wang, L. Wang, Q. Wang, Q. Zhang, F. Cui, G. Jiang, Synthesis of S-type heterostructure π-COF for photocatalytic tetracycline degradation, Chem. Eng. J. 479 (2024) 147534.

[6] J. Cui, L. Ye, X. Chen, J. Li, B. Yang, M. Yang, Q. Yang, D. Yun, S. Sun, Simultaneously promoting adsorption and charge separation in Z-scheme ZnO/Cu_2O heterojunctions for efficient removal of tetracycline, Appl. Surf. Sci. 638 (2023) 15804.

[7] X. Xu, F. Feng, Z. Wan, Y. Wang, M. Yu, X. Han, G. Wu, W. Xing, Rapid electron transfer reinforced by interfacial Co-O bonding in MOF/COF hybrids for highly efficient degrade tetracycline by activating peroxymonosulfate, Colloid. Surface. A 689 (2024) 133686.

[8] Z. Shi, Z. Chen, Y. Zhang, X. Wang, T. Lu, Q. Wang, Z. Zhan, P. Zhang, COF TzDa/Ag/AgBr Z-scheme heterojunction photocatalyst for efficient visible light driven elimination of antibiotics tetracycline and heavy metal ion Cr (VI), Sep. Purif. Technol. 288 (2022) 120717.

[9] X. Guo, D. Yin, K.K. Khaing, J. Wang, Z. Luo, Y. Zhang, Construction of MOF/COF Hybrids for boosting sunlight-induced fenton-like photocatalytic removal of organic pollutants, Inorg. Chem. 60 (2021) 15557–15568.

[10] K.K. Khaing, D. Yin, Y. Ouyang, S. Xiao, B. Liu, L. Deng, L. Li, X. Guo, J. Wang, J. Liu, Y. Zhang, Fabrication of 2D–2D heterojunction catalyst with covalent organic framework (COF) and

MoS_2 for highly efficient photocatalytic degradation of organic pollutants, Inorg. Chem. 59 (2020) 6942–6952.
[11] J. Wang, D. Yin, X. Guo, Z. Luo, L. Tao, J. Ren, Y. Zhang, Fabrication of a covalent organic framework-based heterojunction via coupling with ZnAgInS nanosphere with high photocatalytic activity, Langmuir 38 (2022) 4680–4691.
[12] X. Xu, W. Shao, G. Tai, M. Yu, X. Han, J. Han, G. Wu, W. Xing, Single-atomic Co-N site modulated exciton dissociation and charge transfer on covalent organic frameworks for efficient antibiotics degradation via peroxymonosulfate activation, Sep. Purif. Technol. 333 (2024) 125890.
[13] X. Wang, W. Zhao, B. Ma, S. Qian, Y. Wu, X. Zhang, N.R. Kadasala, Y. Jiang, Y. Liu, A novel all-solid-state Z-scheme $BiVO_4$/Ag/Cu_2O heterojunction: Photocatalytic and photothermal synergistic catalysis of tetracycline under simulated sunlight irradiation, J. Alloys Compd. 1005 (2024) 176191.
[14] X. Meng, L. Yan, M. Wei, T. Wang, T. Xu, Y. Yan, S. Cheng, A novel $Co(OH)_2/Cu_2O$ nanocomposite-activated peroxydisulfate for the enhanced degradation of tetracycline, New J. Chem. 45 (2021) 16705–16713.
[15] Y. Zhou, S. Feng, X. Duan, W. Wu, Z. Ye, X. Dai, Y. Wang, X. Cao, Stable self-assembly Cu_2O/ZIF-8 heterojunction as efficient visible light responsive photocatalyst for tetracycline degradation and mechanism insight, J. Solid State Chem. 305 (2022) 122628.
[16] B. Liu, Y. Wu, J. Zhang, X. Han, H. Shi, Visible-light-driven g-C_3N_4/Cu_2O heterostructures with efficient photocatalytic activities for tetracycline degradation and microbial inactivation, J. Photoch. Photobio. A 378 (2019) 1–8.
[17] J. Niu, Z. Song, X. Gao, Y. Ji, Y. Zhang, Construction of Bi_2WO_6 composites with carbon-coated Cu_2O for effective degradation of tetracycline, J. Alloys Compd. 884 (2021) 161292.
[18] Q. Zhao, J. Wang, Z. Li, Y. Guo, J. Wang, B. Tang, A. Abudula, G. Guan, Heterostructured graphitic-carbon-nitride-nanosheets/copper(I) oxide composite as an enhanced visible light photocatalyst for decomposition of tetracycline antibiotics, Sep. Purif. Technol. 250 (2020) 117238.
[19] Y. Zhu, Y. Pan, E. Zhang, W. Dai. A self-assembled urchin-like TiO_2@Ag–CuO with enhanced photocatalytic activity toward tetracycline hydrochloride degradation, New J. Chem. 44 (2020) 11076–11084.
[20] X. He, H. Fang, D. J. Gosztola, Z. Jiang, P. Jena, W. Wang. Mechanistic insight into photocatalytic pathways of MIL-100(Fe)/ TiO_2 composites, ACS Appl. Mater. Interfaces 11 (2019)

12516−12524.
